# Environmental DNA screening of *Phocoenobacter atlanticus* subsp. *atlanticus* in Atlantic salmon aquaculture

**DOI:** 10.1371/journal.pone.0347930

**Published:** 2026-04-27

**Authors:** David A. Strand, Jannicke Wiik-Nielsen, Saima Mohammad, Hanne Nilsen, Bjarte Langhelle, Matilde Holmeset, Sonal Patel, Hanne Log Persson, Duncan Colquhoun

**Affiliations:** 1 Norwegian Veterinary Institute, Norway; 2 DNV Aquaculture and Ocean Health, Bergen, Norway; 3 University of Bergen, Bergen, Norway; University of Iceland, ICELAND

## Abstract

There has been a drastic increase of pasteurellosis cases in Atlantic salmon aquaculture in Norway since 2018, caused by the bacterium *Phocoenobacter atlanticus* subsp. *atlanticus*, resulting in reduced animal welfare and increased mortalities. Early detection and identification of pathogens are crucial in intensive aquaculture to enable implementation of effective management and mitigation strategies. The increased application of environmental DNA (eDNA) monitoring in aquatic environments demonstrates the potential use for early detection of pathogens in aquaculture. In this study we investigated the usage of eDNA monitoring of *Ph. atlanticus* in Atlantic salmon aquaculture. We conducted a trial to investigate the effect of storage conditions and transport time on eDNA detection of *Ph. atlanticus* from water samples. We then conducted three field trials: a) to investigate the optimal sampling location for eDNA samples from farms with known *Ph. atlanticus* infection status, b) to screen farms presumed non-infected with *Ph. atlanticus* and c) to evaluate the eDNA detection in samples collected from anaesthetic tanks during lice counting. The storage trial revealed a reduction in *Ph. atlanticus* detection after one week at 4 °C or after two days at 22 °C. At farms with known *Ph. atlanticus* infection status we experienced poor detection of *Ph. atlanticus* from water samples collected in the net pen. However, samples collected from treatment water during thermal delousing revealed concentration of *Ph. atlanticus* during treatment, even in farms with negative net pen samples. Subsequent eDNA screening (sampling treatment water) of farms with presumed non-infected status, identified the presence of *Ph. atlanticus* in ~55% of the farms tested. *Ph. atlanticus* was also detected in water samples collected from anaesthetic tanks following lice counting. Our results demonstrate early detection of *Ph. atlanticus,* even in lowly prevalent populations, demonstrating the potential use of eDNA monitoring of pathogens in Atlantic salmon aquaculture.

## Introduction

Worldwide aquaculture production surpassed capture fisheries for the first time in 2022 and further growth is considered essential to meet the rising global demand for aquatic food [1]. Norway is the worlds’ largest producer of farmed Atlantic salmon (*Salmo salar*) with over 1.5 million tonnes sold in 2024 (www.fiskeridir.no). Mortality of salmon during production is, however, high with ~15.4% mortality in the sea phase registered in 2024 [2]. The main causes of mortality are treatment-related injuries following non-medical treatment of salmon lice (*Lepeophtheirus salmonis*) and infectious diseases [2–4]. A significant proportion of this mortality is due to infectious diseases, with pasteurellosis caused by *Phocoenobacter atlanticus* subsp. *atlanticus* [5] representing a considerable challenge in this respect [6,7]. Pasteurellosis causes reduced welfare and losses primarily in Atlantic salmon farmed at sea in Norway and Scotland [6,8]. While incidences of this disease have occurred sporadically since the 1980’s, there has been a drastic increase in the number of outbreaks in Norwegian aquaculture in the period 2018–2024 compared to previous years [2].

Early detection, diagnosis and identification of pathogenic agents, including *Ph. atlanticus,* is important in intensive aquaculture to allow introduction of ameliorating management practices. Environmental DNA (eDNA) and RNA (eRNA), collectively referred to as environmental nucleic acid (eNA) [9], analyses have been used to successfully detect and monitor both micro- and macro-organisms [10–14] including pathogens from aquatic environments [15–19]. While eRNA studies remain scarce [9], there has been a drastic increase in the number of published eDNA studies during the last decade, with a shift from developmental studies towards standard application in biomonitoring [20]. A meta-analysis of eDNA studies found that eDNA methods are generally more cost-effective, more sensitive, and detect a greater number of species than traditional survey methods [21]. Furthermore, molecular approaches (especially PCR and qPCR) provide faster, and more sensitive pathogen detection compared to conventional culture-based techniques [22–24]. The use of eNA in aquaculture has great potential as a surveillance tool for pathogens and other biological threats [25,26]. An example of this is a study by Bernhardt et al. [27] who demonstrated early detection of salmonid alphavirus (SAV) using eRNA monitoring and detection of salmonid alpha virus (SAV) at an earlier stage compared to traditional sampling methods. It has also been found that water sampled from thermal delousing machines, routinely used to remove salmon lice (*Lepeophtheirus salmonis*) from farmed salmon, represents an excellent source of eDNA substrate for detection of *Yersinia ruckeri* infection in Atlantic salmon [28]. The suitability of thermal delousing systems for pathogen detection is most likely due to a combination of stress-related increase in shedding of the pathogen and treatment of large numbers of fish in a limited volume of water, leading to concentration of shed bacterial cells. However, as delousing can be performed using several different methods, and the frequency of such treatment is dependent on lice burden, reliance on thermal delousing-based pathogen surveillance alone has its drawbacks. Norwegian regulations require all sea farms to report lice burdens weekly when the sea temperatures are ≥ 4 °C, or every other week when below 4 °C [29]. To do this, a number of fish, as specified in regulations, are captured, anaesthetized in a holding tank and the number of lice per fish recorded and reported. Thus, we considered the anesthesia tank during louse-counting a possible (and regular) source of eDNA for pathogen surveillance for aquaculture farms using this approach for lice counting.

The goal of the present study was, therefore, to evaluate use of environmental DNA screening of water sampled from thermal delousing machines, and to a lesser extent lice-counting tanks, for detection of *Ph. atlanticus* subsp. *atlanticus* and to investigate whether this type of analysis represents a useful management tool in Atlantic salmon farming.

## Materials and methods

In total four trials were performed, one to test effect of storage conditions and transport time on eDNA detection, while the main study consisted of three trials where sampling was carried out at farm sites. All samples in the field trials were collected by qualified aquaculture health personnel, and no permits were required.

### Sample storage trial

To investigate the effect of storage conditions and transport time on eDNA detection of *Ph. atlanticus* from water samples, a trial with spiked seawater with different bacterial load was conducted. Cultures of the *P. atlanticus subsp. atlanticus* isolate VIO 11693 were grown in liquid medium at room temperature for 20 hours (low bacterial load) or 40 hours (high bacterial load) with shaking at 200 rpm. Optical density (OD_600_) was measured for each culture with a Genesys 10S VIS spectroscopy instrument (Thermofisher). From these cultures, ten-fold dilution series were plated on blood agar with 2% NaCl and incubated for five or six days before counting of colonies. Non sterilised natural seawater was collected at Puddefjorden, Norway, and 500 ml sea water samples were either spiked with 30 ml or 500 ml of bacteria for low and high bacterial load. The spiked samples were stored for 0, 1, 2, 3, 5, 7 or 9 days at either 4 °C or 22 °C before filtration, DNA extraction and qPCR analysis (see below for filtration, DNA extraction and qPCR setup). Additionally, unspiked sea water samples were analysed without storage as a negative control.

### Sampling

The study comprised three field trials, which involved collection of water samples from either (field trial A) the treatment chamber of thermal delousing systems, before, during and following completion of delousing of Atlantic salmon populations and the net pen in which the fish were held; (field trial B) the treatment chamber of thermal delousing systems, before and following completion of delousing of Atlantic salmon populations; or (field trial C) the anaesthetic tank before and after lice counting of Atlantic salmon populations. In all investigations, water samples were taken in sterile, octagonal, 500 ml PET bottles (Corning, Borre, France), refrigerated at ~4 °C and transported overnight to the laboratory within two days of sampling.

### Field trial A

The first field trial was intended to test proof of principle for eDNA detection of *Ph. atlanticus* in water samples collected during thermal delousing from marine Atlantic salmon farms. To achieve this, 16 salmon farms with varying infection status, i.e., clinical outbreak; sub-clinically infected PCR positive fish; suspected infection; uncertain status; and presumed free of infection, were sampled to evaluate the methodology and sampling location (see Table 1). Five water samples were collected from each farm during thermal delousing. One sample was collected downstream of the treated pen before treatment, one sample from the treatment chamber of the thermal delouser before treatment start, one sample from the treatment chamber after 1/3 of treatment time, one sample from the treatment chamber after 2/3 of treatment time and one sample from the treatment chamber after treatment. Samples were collected from a total of 16 farms.

**Table 1:** Summary of eDNA results from trial A, comparing samples from sea net pens and treatment chambers during thermal delousing at 16 Norwegian Atlantic salmon farms with known Pasteurellosis status.

Pasteurellosis state	No. of farms	eDNA detection from net pens	eDNA detection from treatment chambers
**Mild outbreak**	5	1/5	5/5
**Previously positive (PCR), no outbreak**	4	0/4	4/4
**Suspected, but not confirmed**	2	0/2	1/2
**Uncertain status**	2	0/2	2/2
**Control farm, presumed non-infected**	3	0/3	0/3

### Field trial B

In the second field trial, samples were collected from 11 salmon farms in which *Ph. atlanticus* infection was neither diagnosed or suspected, but which were situated within the endemic region and in relative proximity to farms previously diagnosed with *Ph. atlanticus* infection. Six water samples were collected from the treatment chamber from each farm during thermal delousing, three before treatment and three after treatment.

### Field trial C

In the third field trial, two farms with confirmed *Ph. atlanticus* infection status were sampled to evaluate whether water sampled from the anaesthetic tank during weekly lice counting can be used to detect the presence of *Ph. atlanticus*. On each sampling date, three water samples were collected from the anaesthetic tank before lice counting and three samples were collected after. Approximately 60 fish were passed through the anaesthetic tank during the lice counting. Samples were collected on four different occasions from farm C1 and on two different occasions from farm C2.

### Filtration & DNA extraction

Water samples were filtered on arrival at the laboratory or following overnight refrigeration using Nalgene Single Use Analytical Filter Funnels with sterile Cellulose Nitrate filters (0.45µm pore size) attached to a Nalgene Vacuum Manifold and Millipore EZ-Stream pump. Complete samples (500 ml) were filtered or until the filter clogged. In some cases, several filters were required to cope with the high particulate load borne by samples collected late in the delousing procedure. In such cases, the filters were pooled into one sample before DNA extraction. Subsequently, samples with obvious high particulate load were centrifuged at 5000 rpm for 60 min at 15 °C (Sorvall Lynx 6000 centrifuge, Thermofisher). After centrifugation the supernatant was removed and filtered as described above while the pellet was transferred directly to an Eppendorf tube with a 1000 µl pipette. After filtration the filters and pellets (treated individually) were transferred to 5 ml Eppendorf tubes and lysed by adding 370µl ATL buffer (Qiagen) and 30 µl Proteinase K, followed by incubation on a heating block (56 °C) with agitation/shaking overnight. DNA was then extracted with the automated Qiacube system (Qiagen) following the DNeasy blood and tissue protocol and eluded in 200 µl elution buffer.

### PCR analysis

DNA samples were analysed using the primers and probe combination (Patsalm_F TCT AAT ATT GAT GAT CTT GTT TG, Patsalm_R ATT TCC TAA ATT AGG AAA GAT AC) and FAM labelled probe with BHQ-1 quencher (Patsalm_BHQpr ACT TGA TGA AGC TAC ACA ACG TG), described by Sandlund et al. [7] and subsequently confirmed as *Ph. atlanticus* subsp. *atlanticus* specific by Spilsberg et al. [30]. All analyses were run on an AriaMx real-time PCR system (Agilent). Each reaction was performed in a total volume of 20 µl, containing 10 µl Brilliant III Ultra-Fast qPCR master mix, 900 nM forward primer, 900 nM reverse primer, 600 nM probe, and 9.5 µl DNA template. Thermal cycling consisted of a hotstart at 95 °C for 3 min, followed by 45 cycles of denaturation at 95 °C for 5 sec and annealing/extension at 57 °C for 10 sec. Negative and positive controls were included with each qPCR run. Reactions suspected of PCR inhibition were re-analysed with undiluted and x10 diluted DNA template. Samples with Cq value < 40 were scored as positive, while samples with a Cq value ≥ 40 were scored as negative for trial A and trial B (S1 Table). Unfortunately, Cq values of 33.64 and above, from negative control samples during analysis of samples collected in trial C, indicated contamination during the automated DNA extraction step (QIAcube). In this case, to account for this contamination, a stringent cutoff was used to evaluate the results and samples with Cq value ≤30 were scored as positive, while Cq value > 30 were scored as negative (S1 Table).

### Statistical analysis

To evaluate differences in detection of *Ph. atlanticus* from eDNA samples from the different sampling sites in trial A and trial B, we performed a logistic regression (binominal GLM) with a post-hoc pairwise comparison using Estimated Marginal Means (EMMs). For eDNA samples from trial C, a stringent cutoff at Cq value 30 was used due to contamination and consequently all samples from farm C2 were scored negative. Samples collected before and after lice counting from farm C1 were compared with a Fisher’s exact test as samples collected before treatment were all scored as negative. All statistical analyses were performed with R (4.4.1) and RStudio (2024.04.2 + 764).

## Results

### Sample storage

**Fig 1:**
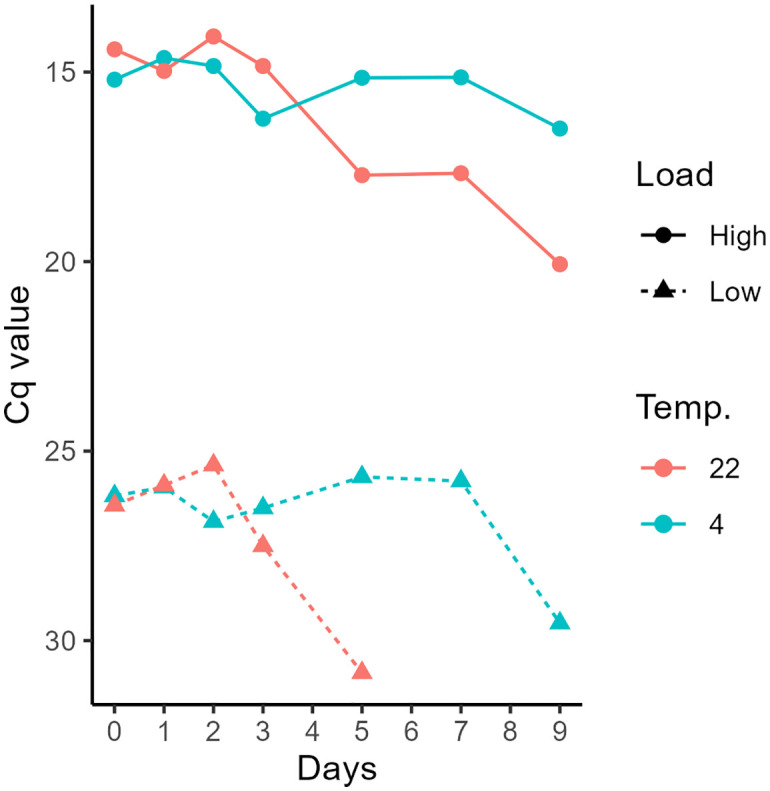
Samples storage trial results. Line plot of Cq values (inverted y-axis) from *Ph. atlanticus* subsp. *atlanticus* qPCR analysis from spiked sea water samples with low and high bacterial load, stored either at 4 °C or 22 °C for up to nine days.

The optical density (OD_600_) for the low and high load cultures was 0.002 and 0.188, and the CFU/ml was estimated to ~1,4 x 10^5^ and ~1,2 x 10^6^, respectively. The sea water samples without added bacterial culture (control) did not amplify for *Ph. atlanticus* (S2 Table). For samples spiked with a high load of bacterial culture, the Cq values were relatively stable throughout the period of nine days for the samples stored at 4 °C, while there was an increase in Cq value after day three for the samples stored at 22 °C (Fig 1), indicating a reduction of *Ph. atlanticus* DNA in the water. For samples spiked with a low load, stored at 4 °C, the Cq values were relatively stable until day seven followed by an increase in Cq value to day nine (Fig 1). For the spiked samples with a low load, stored at 22 °C, the Cq values increased after two days with no amplification at day seven or day nine. The qPCR results for the storage trial are reported in S2 Table).

### Field trial A

**Fig 2:**
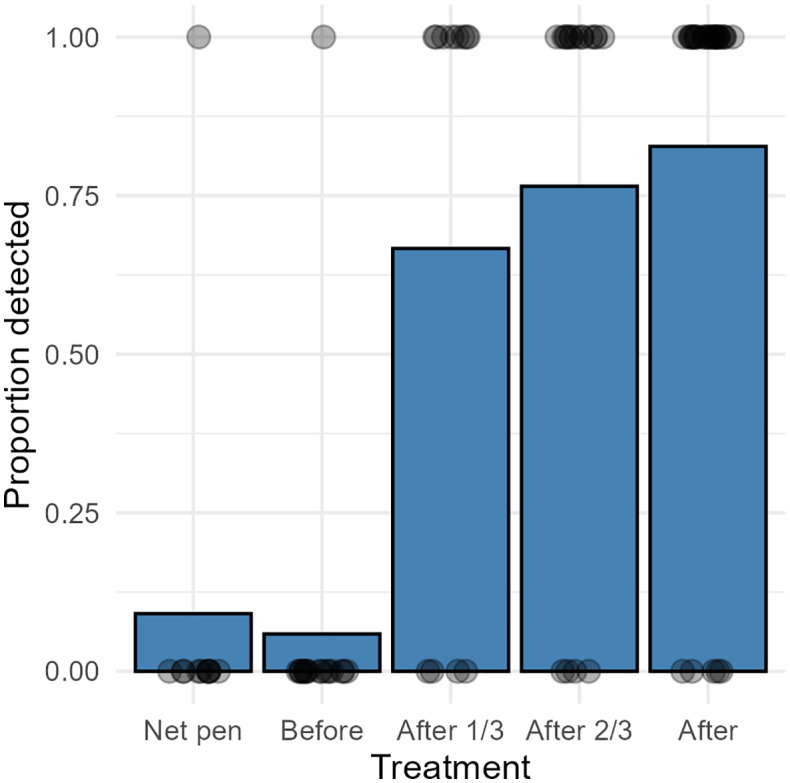
eDNA samples field trial A. A bar plot showing proportion of detections from *Ph. atlanticus* subsp. *atlanticus* qPCR analysis of eDNA samples from field trial A where samples were collected from Norwegian Atlantic salmon farms with known pasteurellosis status. Individual points (circles) represent raw observations (0 = not detected, 1 = detected), jittered horizontally for visibility. Water samples were collected from pen and treatment chamber (before, after 1/3, after 2/3 and after full thermal treatment). Only results from farms where one or more samples were PCR positive for *Ph. atlanticus* subsp. *atlanticus* are included. Detections from filters and pellet are included.

The qPCR results with Cq values from trial A, B and C are provided in S1 Table. For trial A, *Ph. atlanticus* was detected in eDNA sampled from 12 of the 16 farms with ‘recognised’ *Ph. atlanticus* infection status (Table 1). Water samples taken from the net pens within which the fish were held were all PCR negative, with the exception of a single net pen in the ‘confirmed outbreak’ group. Thermal delousing-based sampling proved much more sensitive with the bacterium being detected in all farms from the ‘confirmed outbreak’ and ‘subclinically infected, PCR positive fish’ sites (Table 1). The bacterium was also detected in three of four farms with ‘suspected’ (1 of 2) and ‘uncertain’ (2 of 2) infection status. None of the three farms that were considered non-infected tested positive for *Ph. atlanticus* (Table 1). There was a significant difference (Binomial GLM p < 0.001, S3 Table) in the proportion of *Ph. atlanticus* detected from eDNA samples collected in the net pet and thermal chamber compared to the samples collected during and after treatment (Fig. 2). The post-hoc test (EMMs) revealed a significant increase in detection in eDNA samples collected from the treatment chamber during and after thermal treatment compared to samples collected before treatment (Fig 2, S4 Table).

### Field trial B

**Fig 3:**
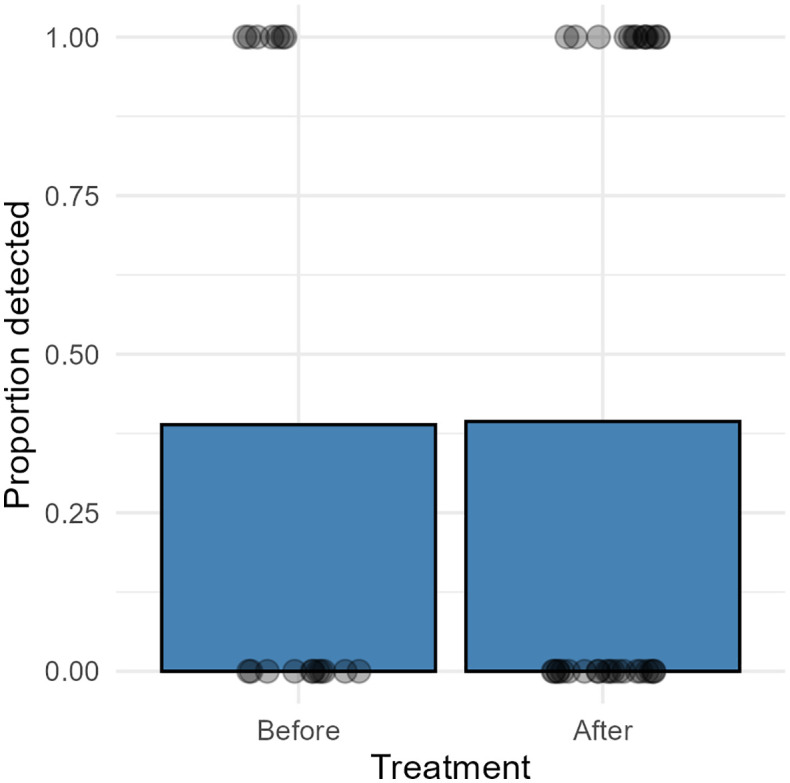
eDNA samples field trial B. A bar plot showing proportion of detections from *Ph. atlanticus* subsp. *atlanticus* qPCR analysis of eDNA samples from field trial B where samples were collected from Norwegian Atlantic salmon farms with no previous history of Pasteurellosis. Individual points (circles) represent raw observations (0 = not detected, 1 = detected), jittered horizontally for visibility. Water samples were collected from the treatment chamber before and after thermal treatment. Only results from farms where one or more samples were PCR positive for *Ph. atlanticus* subsp. *atlanticus* were included.

In field trial B, *Ph. atlanticus* was detected in eDNA samples collected during thermal delousing from 6 of the 11 farms previously considered as infection free (Table 2). The Cq values of the eDNA samples from these farms were on average higher (i.e., lower bacterial load) than those identified from the farms with confirmed *Ph. atlanticus* infection in field trial A (S2 Table). There were no significant statistical differences (Binomial GLM p = 0.972) in proportion of detection between samples collected before or after treatment in trial B (Fig 3). Of the six farms that were confirmed positive for *Ph. atlanticus* eDNA in this trial, fish from four of the farms subsequently tested positive for *Ph. atlanticus* seven weeks to four months post eDNA sampling with at least one developing clinical disease (Table 2). Of the five farms that tested negative for *Ph. atlanticus* eDNA, fish from three of these farms tested positive for *Ph. atlanticus* infection three to six months post eDNA sampling (Table 2).

**Table 2:** Summary of eDNA results from field trial B. Screening of samples from Norwegian Atlantic salmon farms presumed non-infected with *Ph. atlanticus*. The clinical state post eDNA analysis was evaluated and reported by individual farm fish health personnel.

Fish farm #	Pasteurella eDNA detection	Clinical state post eDNA analysis
1	Negative	Fish test PCR positive 5 months later
2	Negative	Pasteurellosis detected 5–6 months later
3	Negative	Pasteurella-free at slaughter 3 month later
4	Negative	Pasteurella-free 6 months later
5	Negative	Some fish PCR positive (growth test) 3 months later
6	Positive	No sign of pasteurellosis 6 months later
7	Positive	No sign of pasteurellosis 5 months later
8	Positive	Pasteurellosis confirmed (growth test) 7 weeks later
9	Positive	Fish test PCR positive 4 months later
10	Positive	Fish test PCR positive 2 months later
11	Positive	Fish test PCR positive 2 months later

### Field trial C

**Fig 4:**
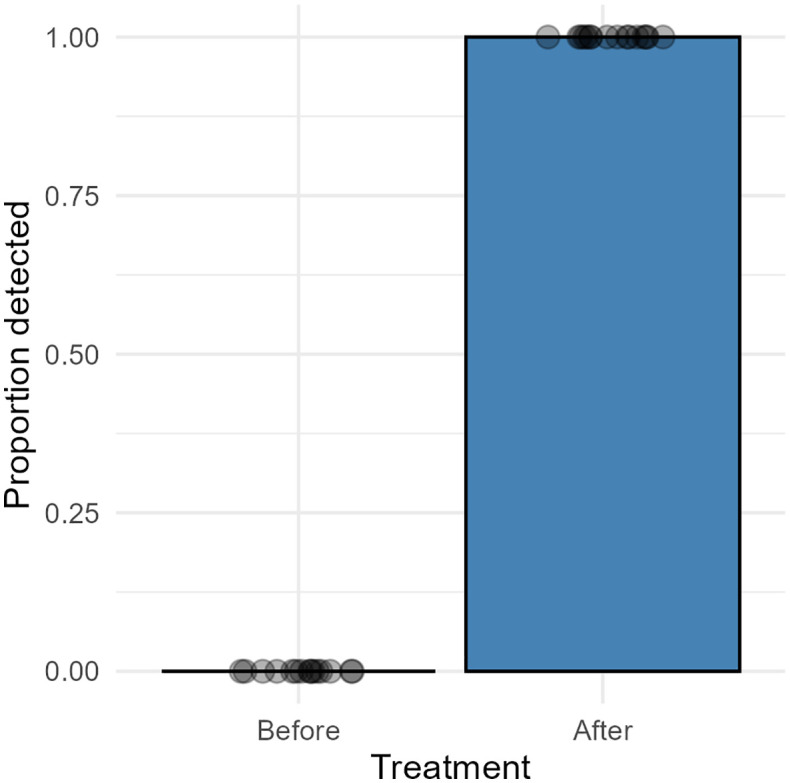
eDNA samples field trial C. A bar plot showing proportion of detections of *Ph. atlanticus* subsp. *atlanticus* qPCR analysis from eDNA samples from field trial C. Individual points (circles) represent raw observations (0 = not detected, 1 = detected), jittered horizontally for visibility. Samples were collected from the anaesthetic tank from two Norwegian Atlantic salmon farms with diagnosed *Ph. atlanticus* infection, before and after lice treatment. Only results from farm C1 were included in figure as all samples from farm C2 were scored negative for *Ph. atlanticus*. Due to contamination of samples during the automated DNA extraction (Qiacube) samples with Cq value ≤ 33 were scored as PCR positive, while samples with a Cq value > 33 were scored as negative.

The PCR analyses/results generated in trial C were scored more strictly due to cross-sample DNA contamination in the automated QIAcube extractor. All samples from field trial C may have been affected, as these were extracted at the same time-period in 2024. The samples from field trial A and B were extracted in 2022 and 2023 and there was no sign of contamination of those samples. The negative controls (extraction control of the automated Qiacube) amplified with Cq value 33.64 and above. Taking this into account, only samples from farm C1 could be considered as secure PCR positive for *Ph. atlanticus*, demonstrating a significant increase (Fisher test, p < 0.001) in pathogen detection in the anaesthetic tank following lice counting (Fig 4, S2 Table).

## Discussion

Environmental DNA monitoring has the potential to improve early detection of pathogens in aquaculture [25,31], and here we demonstrated successful detection of *Ph. atlanticus* subs *atlanticus* in eDNA samples collected from farms with and without recognised Pasteurella issues. While the bacterium was only detectable from the net pen water in which the fish were maintained, we demonstrated high levels of detection, in both overtly and covertly infected fish populations, from water samples collected from the treatment-chamber of thermal delousing machines during and following treatment of fish. While sampling from anaesthetic tanks during weekly lice counting was not extensively tested, it did enable detection of *Ph. atlanticus* in overtly infected fish (trial C).

The initial trial with *Ph. atlanticus* spiked water samples demonstrated the possibility to store the samples for up to seven days at 4 °C before seeing a reduction in qPCR detection (increased Cq value) compared to two days at 22 °C. These results are similar to the findings in a study by Hinlo et al. [32] that showed higher loss of eDNA concentration in water samples stored at room temperature compared to samples stored at refrigerator temperature and recommend short term storage (up to 3–5 days) at low temperature when filtration within 24 hours is not possible. Several factors influence eDNA degradation [33–35] and degradation of eDNA is higher with increased water temperature [36,37]. Yamanaka et al. [38] showed that benzalkonium chloride (BAC) at a final concentration of 0.01% was effective reduce eDNA degradation even at ambient temperatures with a retention of up to 50% for 10 days storage. While our results showed no reduction in detection for up to seven days storage at 4 °C, our samples were spiked with bacteria culture and most likely consisting of intracellular DNA and would be detectable for longer compared to extracellular eDNA, e.g., smaller eDNA fractions (e.g., extracellular eDNA) will degrade faster that larger eDNA fractions (e.g., intracellular eDNA) [39]. As the water samples collected in this study were stored at refrigerate temperatures and shipped over night and filtered with 24 hours of receiving the samples, it is unlikely that this affected the detection likelihood of *Ph. atlanticus*.

Swab samples from salmon skin and/or gills for qPCR screening of *Ph. atlanticus,* also initially tested in trial A, were quickly excluded from the study due to the low detection rate compared to treatment-chamber results (data not shown). That swabbing of individual fish identified relatively few PCR positives in most cases, is consistent with observations from the field indicating that the prevalence of infection in individual fish may be extremely low in the case of early infections. It also highlights one of the main strengths of eDNA-based detection methodology in that it tests the whole population rather than individual fish. During validation of the eDNA method (field trial A) *Ph. atlanticus* was only detected in a single incidence from samples taken from the open net pens in which the infection had been previously diagnosed. This could conceivably be related to a general low prevalence of infection, low bacterial shedding by the fish under relatively low stress conditions in the net pen, dilution caused by currents and the vast volume of water present, or any combination of these factors. While the expected shedding of pathogens is usually high and easily detectable during acute disease episodes, shedding associated with latent infections is expected to be considerably lower and harder to detect with eDNA monitoring. During an active outbreak of yersiniosis in a marine salmon farm, with substantial associated mortality, we found *Y. ruckeri* detectable in sub-surface water samples up to 100 meters downstream of the affected net pen, but not at 200 or 500 meters [28], suggesting rapid dilution of shed bacterial cells. Although the analyses were not strictly quantifiable, we demonstrated Cq values correlating with a steadily rising number of *Ph. atlanticus* cells within the treatment chamber as treatment progressed (Fig 2). Riborg et al. [28] demonstrated in a laboratory trial that shedding of pathogenic bacteria (in that case *Y. ruckeri*) by sub-clinically infected fish increased during thermal delousing treatment, compared to shedding in the tank in which they had previously been held. A similar increase in stress-related shedding of *Ph. atlanticus* may have occurred in the present study, but the increasing number of bacterial cells within treatment chambers in the present study could conceivably be simply related to the constantly increasing number of fish treated in a limited volume of water. Thus, the concentration of shed bacterial cells following treatment of a large number of fish in a restricted water volume makes thermal delousing treatment chambers ideal for eDNA sampling [28]. In a few cases, eDNA samples collected from the treatment chamber prior to treatment were also PCR positive for *Ph. atlanticus* DNA (Figs 2–3). This does not necessarily mean the presence of viable bacteria, as all machinery moving from farm to farm is subject to mandatory disinfection, but it may indicate that treatment chamber was not completely clean.

*Ph. atlanticus* was detected in six of the eleven Atlantic salmon farms in trial B, in which there were no prior indications or suspicions of *Ph. atlanticus* infection. This suggests that the infection may be more widespread than the number of diagnosed clinical outbreaks suggests. However, the positive sites only showed trace amount of *Ph. atlanticus*, and there was no clear increase in detection after thermal treatment, suggesting very low prevalence in these salmon populations. At four of these six eDNA positive farms, *Ph. atlanticus* infection was subsequently confirmed in fish tissues by PCR, two to four months after eDNA detection. These results support the concept of using eDNA monitoring for early detection of pathogens in aquaculture [27,40]. Detection of *Ph. atlanticus* in a population of fish does not, however, appear to necessarily result in manifestation of clinical disease, as this was not observed in two of the farms with *Ph. atlanticus* detection, six and seven months respectively following eDNA detection. Further, *Ph. atlanticus* was detected three to six months later in three of the five farms that tested negative for *Ph. atlanticus* during the eDNA screening. Our results cannot reveal whether the collected eDNA samples represented false negative samples or if *Ph. atlanticus* was introduced into the farms in between the eDNA sampling and the detection of the bacteria several months later. Further, while a single eDNA sampling might detect low prevalent pathogens during thermal treatment, in our case it was not sufficient to predict future pasteurellosis cases. Ideally, eDNA sampling with frequent intervals would increase the likelihood for detection, reveal changes in prevalence and provide more robust data for early decision-making and mitigations efforts.

Despite the apparent advantages of thermal delousing-based sampling for pathogen detection, there are, however, increasing concerns in the industry regarding its use and negative effects on fish welfare. Thermal treatment has been directly related to both acute and delayed mortalities associated with the treatment itself [3,4,41,42]. Stige et al. [43] also identified a positive statistical association between thermal delousing and subsequent *Phocoenobacter* infection post-treatment. It is, therefore, quite possible that thermal delousing will be restricted in the future. One further disadvantage is that delousing treatments are poorly suited to regular health monitoring as the frequency of delousing is dependent on the louse burden at any particular time. In contrast, weekly salmon lice counting is mandatory in Norwegian salmon farming, and although not extensively tested in the current study, we did find that eDNA samples taken from the anaesthetic baths used during lice counting (field trial C) offer the potential for detection of *Ph. atlanticus* and that this source of sampling should be investigated further. We did experience contamination during the automated DNA extraction of samples from field trial C, and we applied a stringent interpretation of those results. This also highlights the importance of using negative extraction controls to reveal potential contamination. In conclusion, we found eDNA analysis to be an effective non-fatal methodology for surveillance of *Ph. atlanticus* infection in sea-farmed Atlantic salmon in Norway, prior to eventual outbreak of disease. Implementation of routine eDNA monitoring in aquaculture can improve biosecurity by early detection of relevant pathogens, allowing for early decision-making and mitigations efforts.

## Supporting information

S1 Table Overview of eDNA samples and qPCR results. (XLSX)

S2 Table Overview of the results from the storage trial where seawater was spiked with *P. atlanticus.* (XLSX)

S3 Table Summary of the binomial GLM results for the effect of sampling location on eDNA detection at trial A. (XLSX)

S4 Table Results of post-hoc analysis using Estimated Marginal Means (EMMs) comparing proportion of detection from the different locations in trial A. (XLSX)
